# Suffering a Loss Is Good Fortune: Myth or Reality?

**DOI:** 10.1002/bdm.2056

**Published:** 2017-11-29

**Authors:** Cui‐Xia Zhao, Si‐Chu Shen, Li‐Lin Rao, Rui Zheng, Huan Liu, Shu Li

**Affiliations:** ^1^ School of Psychology Shandong Normal University Jinan China; ^2^ CAS Key Laboratory of Behavioral Science Institute of Psychology, Chinese Academy of Sciences Beijing China; ^3^ Department of Psychology University of Chinese Academy of Sciences Beijing China; ^4^ Centre for Mental Health Nanchang University Nanchang China

**Keywords:** suffering a loss, anecdote‐based scale, Socioeconomic Index, subjective well‐being

## Abstract

We sometimes decide to take an offered option that results in apparent loss (e.g., unpaid overtime). Mainstream decision theory does not predict or explain this as a choice we want to make, whereas such a choice has long been described and highly regarded by the traditional Chinese dogma “吃亏是福” (suffering a loss is good fortune). To explore what makes the dogma work, we developed a celebrity anecdote‐based scale to measure “Chikui” (suffering a loss) likelihood and found that:(i) people with higher scores on the Chikui Likelihood Scale (CLS) were more likely to report higher scores on subjective well‐being and the Socioeconomic Index for the present and (ii) the current Socioeconomic Index could be positively predicted not only by current CLS scores but also by retrospective CLS scores recalled for the past, and the predictive effect was enhanced with increasing time intervals. Our findings suggest that “suffering a loss is good fortune” is not a myth but a certain reality. © 2017 The Authors Journal of Behavioral Decision Making Published by John Wiley & Sons Ltd.

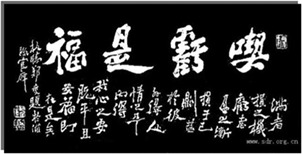
“吃亏是福——满者损之机,亏者盈之渐。损于己则益于彼,外得人情之平,内得我心之安,即平且安,福即在是矣。”

吃亏是福 (suffering a loss is good fortune)

——Zheng Banqiao/郑板桥 (1693–1765)

## Introduction

The ability to make decisions and carry out effective actions for achieving rewards and avoiding punishments is central to intelligent life (Schall, 2001). In the real world, many decisions on important life events are to accept or reject an offered **single option** such as straightening of teeth, admission to college, counterfeit cash, a marriage proposal, a home mortgage, early retirement, or signing away an inheritance.

When deciding whether to take an option, quite often in real life, the offered option will result in apparent loss such as the earlier mentioned “unpaid overtime” or “signing away an inheritance”. In such a case, to reject an option resulting in loss is exactly what the “Law of Survival” wants us to do. The “should‐do” behavior has been well documented in both Chinese and Western literature. For instance, Kuan‐tzu said “no one can resist advantages or will embrace disadvantage voluntarily (Kuan‐tzu, 740‐645 BC, p.1074–1075).” In *The Wealth of Nations*, published in 1776, Adam Smith famously argued that economic behavior was motivated by self‐interest (Ashraf, Camerer, & Loewenstein, 2005).

In fact, however, we did choose to accept an offered loss ourselves in daily activities. That is, we accepted an offer of “unpaid overtime,” “innocent being used,” or “being falsely accused and condemned” from time to time. Does the choice of an offered loss represent a decision bias or a deliberate decision? Due to a lack of a pertinent model or theory available to explain the mechanisms producing decision outcomes, little is known about why we choose to take an option that results in loss.

However, a long‐lasting Chinese dogma, “吃亏是福” (suffering a loss is good fortune), a phrase attributed to the multitalented poet‐calligrapher Zheng Banqiao (Hammond, 2007), seems to depict, explain, and value such action of taking an option that results in loss. Even today, the old dogma is still heard. For example, we can easily find several books on the market entitled *Suffering a Loss is Good Fortune* (J.H. Liu, 2011; Zhao, 2008). There is a family drama TV series entitled *Suffering a Loss is Good Fortune*, directed by He Qun, and an American comedy‐drama film *Someday This Pain Will Be Useful to You*, directed by Roberto Faenza and based on Peter Cameron's novel of the same name, it is translated into Chinese as “吃亏是福” (Chikui shi fu).

Interestingly, not only the media are enthusiastic advocates of telling the story of “suffering a loss is good fortune;” people from all walks of life are willing to believe in this story. Our preliminary investigation found that members of young generations or older generations tended to agree with the dogma “suffering a loss is good fortune.”

The next question we might ask is why the old belief can be passed on from generation to generation. In particular, why is “Chikui” a choice people want to make? Could it be real gold or fool's gold?

In classical decision theory, options are presented as points in a multidimensional space, in which each dimension represents a distinct attribute that describes the object (Méndez, 1974; Birnbaum, 1997; see also Sun, Li, Bonini, & Su, 2012). As such, a single option that results in loss can also be presented as a unique point in a multidimensional space. With this in mind, it would be easier for us to understand why we should or why we should not choose the “Chikui” option.

To put it normatively, when options are represented by a fixed set of dimensions, choices are assumed to be guided by the principle of value maximization (Luce, 1959). Each option *x_i_* is assigned a value *v*(*x_i_*), such that the decision maker selects the option with the highest value in the face of a given set of dimensions (Tversky & Shafir, 1992).

**Figure 1:**
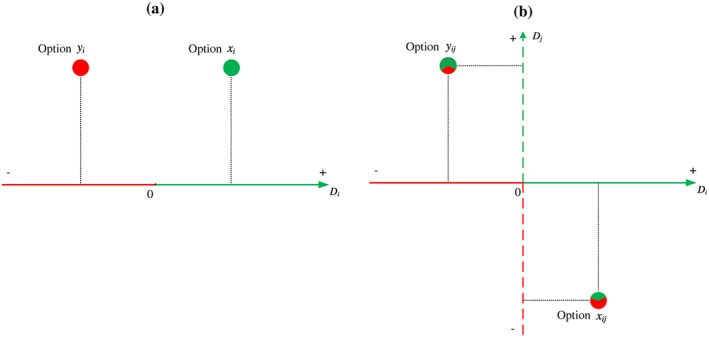
Option‐representing framework for explaining Chikui choice. (a) D_*i*_ = offered money, goods, time, health, opportunity, relationship, *mianzi*, or somewhat other dimension. If Option *x_i_* or Option *y_i_* is a single offered option that needs to be accepted or not, then Option *x_i_* should be accepted while Option *y_i_* should be rejected because *v*(*x_i_*) is always positive while *v*(*y_i_*) is always negative. If Option *x_i_* and Option *y_i_* constitute a pair of offered options from which to choose, then, according to the principle of value maximization (Luce, 1959), Option *x_i_* should be selected because *v*(*x_i_*) > *v*(*y_i_*). (b) D*i* = offered money, goods, time, health, opportunity, relationship, or *mianzi* dimension; D*j* = any extra dimension that is not offered but self‐generated. If Option *x_ij_* or Option *y_ij_* is a single offered option that needs to be accepted or not, then Option *x_ij_* should not always be accepted while Option *y_ij_* should not always be rejected, because *v*(*x_ij_*) is NOT always positive while *v*(*y_ij_*) is NOT always negative [Colour figure can be viewed at http://wileyonlinelibrary.com]

If we define “Chikui” (suffering a loss) as choice behaviors that result in an apparent loss in terms of money, goods, time, health, opportunity, relationship, and even *mianzi* (face), then, all these could serve as offered dimensions on which an offered loss is represented. That is, a single option (Option *y_i_*) that results in loss can be presented as a unique point by the decision maker in a one‐dimensional or in multidimensional space. This will render the understanding of the single option choice much simpler: all we need to do is to accept the option that results in gain (Option *x_i_*) and reject the option that results in loss (Option *y_i_*) (see Figure 1a).

It is obvious that to reject Option *x_i_* or to accept Option *y_i_* is illogical according to economic rationality models (Delton, Krasnow, Tooby, & Cosmides, 2011) and cannot be understood and communicated from the viewpoint of value maximization (Luce, 1959). Moreover, “Chikui” (taking Option *y_i_*) seems to contradict the conventional view of *loss aversion*, which is commonly interpreted as the (marginal) disutility of a given loss being larger than the (marginal) utility of the same amount of gain (Kahneman & Tversky, 1979). Considering that the existing decision theory sees “maximizing gains” or “minimizing losses” as its basic principle, those theories will not predict or explain “Chikui” (suffer a loss) as a choice we want to make. As thus, conventional decision theory does not account for a “Chikui” choice above and beyond the law of the jungle, which is “achieving rewards and avoiding punishments.” In other words, the incremental contribution that conventional decision theory makes to explain why the offered loss is frequently selected by ordinary people is limited.

It is worth noting, however, that if there exists any extra dimension, D_*j*_, that is not passively offered by the proposer but is actively and creatively produced by the decision maker to present the offered option per se, then making a loss choice (suffer a loss) can be easily understood and communicated. Namely, with the extra dimension being generated and a delayed value (utility) being assigned to the generated dimension, *v*(*x_ij_*) is NOT always positive while *v*(*y_ij_*) is NOT always negative. The value of an offered loss (*v*(*y_ij_*)) can turn out to be positive if the value (utility) assigned to the option on the newly generated dimension (D_*j*_) is greater than that assigned to the option on the offered dimension (D_*i*_) (Figure 1b).

To say that “actively generating an extra dimension and then assigning a delayed value (utility) to the self‐generated dimension D_*j*_” means that “people subjectively believe they will gain later good fortune rewarded by an unpredictable life situation” (i.e., a person believes that such a *subjective* later good fortune might exist). To test our conjecture, it is essential for us to find and present evidence that after suffering a loss, there does exist such a later good fortune (gain) rewarded by an unpredictable life situation (self‐generated dimension D_*j*_) (i.e., such an *objective* later good fortune (gain) does exist).

If such an *objective* later good fortune (gain) does exist, then it can be taken as empirical evidence that there must be some self‐generated dimension on which the good fortune (gain) was assigned to the option. Otherwise, taking an option that results in loss appears to be completely unreasonable or not logical. The study reported in this paper therefore intended to collect empirical evidence regarding the relationship between “suffering a loss” and “good fortune.” The study is organized as follows.

In Study 1, we searched for “Chikui”‐related anecdotes told by worldwide celebrities, and we then used these anecdotes as critical incidents of successful persons or winners to develop an anecdote‐based scale to measure “Chikui” likelihood. That is, we aimed to develop a reliable and valid Chikui Likelihood Scale (CLS) that has shown criterion‐related, empirical, convergent, discriminant, and incremental validity. In Study 2, we investigated whether there is a linear correlation between “Chikui” likelihood and real benefits in a real‐world setting using the newly developed CLS. In Study 3, we investigated the possibility that “Chikui” likelihood has a reverse‐predictive effect on later material or mental benefits. That is, we examined whether retrospective “Chikui” likelihood can predict a person's long‐term material or mental benefit.

## Study 1: Developing an Anecdote‐based Scale to Measure “Chikui” Likelihood

### Part 1

Study 1 involved two parts. The first involved a development of the CLS and provided initial reliability and validity data. The second focused on providing further evidence on validity of the CLS.

### Method

#### Identification of “Chikui”‐related anecdotes

If “good fortune” is seen as a way of being “successful in life,” the dogma that “suffering a loss is good fortune” would suggest that “suffering a loss” (Chikui) is very likely to be what people need to do to be “successful” and can be identified and defined in behavioral terms.

Accordingly, we searched for Chinese and foreign anecdotes of celebrities (successful people) in history, culture, literature, politics, science, and business.

Ten psychology graduate students taking a decision‐making course independently read through anthologies of celebrity anecdotes and identified those “behaviors selected by someone that result in an apparent loss of money, goods, time, health, opportunity, relationship, and even *mianzi* (face).” Hundreds of anecdotes were considered. Although the final selection of “Chikui”‐related anecdotes may not be exhaustive, it can be considered fairly representative.

Disagreements in raters' selections were resolved by discussion. The same individuals then independently rated each selected anecdote on two dimensions: item clarity and response appropriateness. Raters were blind to the purpose and predictions of the study.

The following numbers of anecdotes were classified as “Chikui”‐related: three of the approximately 24 anecdotes in *Twenty‐Four Filial Deeds* (Chen & Smith, 2010), five of the approximately 66 anecdotes in *Li Ka‐shing's Complete Biography* (Sun, 2010), 29 of the approximately 200 anecdotes in *Stories of World Famous Celebrities* (Cui, 2010), two of the approximately 19 anecdotes in *First Families* (Y.Z. Liu, 2011), and 15 of the approximately 141 anecdotes in *Anecdotes of Celebrity* (Ma, 2000). Example anecdotes included those related by Li Ka‐shing, Wang Lo Kat, the Rockefeller Family, Loo‐Keng Hua, and Mahatma Gandhi in India.

#### Generation and selection of “Chikui”‐related scenarios

A total of 54 anecdotes were identified as being related to “Chikui.” We then rephrased each anecdote into a brief scenario with two options (Option A: the person described in the scenario chooses “Chikui;” Option B: the person described in the scenario does NOT choose “Chikui”) for respondents. Fifteen graduate students studying psychology were then asked to choose from the two options for each scenario. According to a binomial distribution, if more than 10 of the 15 evaluators selected Option A, the scenario was retained as satisfying the relevant criteria. Otherwise, the scenario was eliminated from consideration (Siegel, 1956).

Accordingly, 27 scenarios were eliminated. Another three scenarios were also eliminated because a group of four scenarios told a similar story.

As a result, a total of 24 scenarios (Appendix S1) were generated by rewriting the anecdotes of celebrities to serve as the items of the CLS. Following the logic of “walking in another person's shoes,” we instructed the respondents to respond to each scenario as follows: *Please carefully read the following scenarios and*, *based on your personal experiences*, *enter values between 0% and 100% to indicate the likelihood that you would act in the same way that is described*. A greater value indicates greater likelihood that the respondent chooses “Chikui.” The following is an example drawn from the 24 scenarios:

Zhang went to a store to purchase some goods. After Zhang paid for the goods and went home, a representative from the store called and told Zhang that one of the 100‐yuan bills was a counterfeit note, but the person was unsure whether the bill came from Zhang. Nevertheless, Zhang returned to the store and exchanged the money.

Imagine that you were Zhang in this situation. What is the likelihood that you would do the same thing? ( ) 0%________100%

#### Participants and procedure

To develop the CLS using a geographically convenient sample, a total of 671 (433 female, 65.80%) adult passengers departing from Ji'nan West Railway Station were interviewed in the waiting lounge between 2013 and 2014. These passengers came from all walks of life (approximately 60 occupations) and from 19 different provinces in China, and ranged from 18 to 74 years of age (*M* = 31.30, *SD* = 9.48). Each participant completed the scales independently and was given a small gift (worth approximately 15 RMB yuan) for his/her participation.

The 671 participants were randomly divided into two groups by spss statistical software. Exploratory factor analysis was performed on 335 participants, and confirmatory factor analysis (CFA) was performed on the other 336 participants. Differences between the two groups in age (*t* = 0.24, *p* > 0.05), gender (χ^*2*^ = 1.03, *p* > 0.05), years of education (*t* = 1.01, *p* > 0.05), and monthly income (*t* = 1.69, *p* > 0.05) were insignificant. All 671 participants were subjected to an analysis of internal consistency (Cronbach's alpha) of the CLS.

A sample of 92 undergraduates (58 females, 63.04%) who participated in a test–retest reliability study was recruited from the School of Psychology in Shandong Normal University. They participated for monetary compensation (¥5).

Two‐hundred undergraduate students (78 women, 122 men) who participated in a validation study (criterion‐related and empirical validity) were recruited from the School of Life Science in Shandong Normal University. They participated for monetary compensation (¥5).

All items were coded and scored, and all data were entered, checked for missing values, and analyzed using the statistical programs spss version 13.0 and Amos version 7.0.

### Results and discussion

#### Item discrimination

In the assessment of item discrimination, the discrimination index (D) was computed by subtracting the mean score of participants in the lower group (27%) from the mean value of those in the upper group (27%) and dividing it by the maximum possible discrimination. A value of 0.19 or below indicated that the item was subject to improvement (Hopkins, 1998). Items with D < 0.19 included items 3, 4, 8, 13, 14, and 15. These six items were eliminated from the scale, thereby reducing the number of items from 24 to 18.

#### Factor structure

##### Exploratory factor analysis

The Kaiser‐Meyer‐Olkin measure of sampling adequacy and the Bartlett Test of Sphericity were conducted on the data before factor extraction to ensure that the characteristics of the data set were suitable for factor analysis. The analysis yielded an index of 0.84 in concert with a highly significant Bartlett Test of Sphericity (χ^2^ = 1039.00, *df* = 153, *p* < 0.001). To determine the number of factors underlying “Chikui,” a principal component analysis using oblique factor rotation (promax) was conducted on the 18 items of the CLS.

The retention of factors was determined by several criteria. First, the Kaiser (1961) criterion of eigenvalues greater than 1 indicated a four‐factor solution. Second, scree plot analysis (Cattell, 1966) suggested a three‐factor solution. Third, we ran the parallel analysis procedures (Horn, 1965), which Zwick and Velicer (1986) found outperforms other methods such as the Kaiser criterion and scree plot, and identified a three‐factor solution.

Inspection of the Kaiser's rule, the scree plot analysis, and the Horn's parallel analysis showed that three factors underlie the 18 items of the CLS. The following criteria were used to determine whether an item loaded on its underlying factor: (i) the item had to have a factor loading of 0.40 or better on one factor and (ii) the cross‐loading differential across the two factors had to be less than 0.20. Items 5, 6, 18, 20, 21, and 22 were eliminated because of the criteria mentioned earlier. Finally, principal components analysis with oblique rotation was conducted with the remaining items, resulting in a three‐factor solution that explained 46.47% of the variance.

The model's factor structure is shown in Table 1. The first factor included five items (Chikui for conscience) that explained 27.56% of the variance, the second factor included four items (Chikui for wealth) that explained 10.49% of the variance, and the third factor included three items (Chikui for reputation) that explained 8.42% of the variance.

**Table 1:** Factor loadings of the 12 items of the Chikui Likelihood Scale

Items	Factor 1	Factor 2	Factor 3
Chikui for conscience			
Item 23	**0.72**	0.11	0.18
Item 24	**0.68**	0.10	0.12
Item 7	**0.64**	−0.08	0.16
Item 9	**0.56**	0.17	0.07
Item 16	**0.52**	0.23	0.04
Chikui for wealth			
Item 11	−0.00	**0.68**	0.28
Item 19	0.14	**0.67**	−0.18
Item 17	0.38	**0.54**	−0.05
Item 12	0.09	**0.52**	0.39
Chikui for reputation			
Item 2	0.25	−0.07	**0.63**
Item 1	−0.01	0.06	**0.63**
Item 10	0.27	0.15	**0.62**

*Note*: Loadings greater than or equal to 0.40 are shown in bold.

##### Confirmatory factor analysis

To confirm the factor structure found in exploratory factor analysis, we performed a CFA on the 12 items of the CLS to determine whether a three‐factor solution best fit the data compared with alternative models of one‐factor solutions. The maximum likelihood estimation procedure was chosen to assess the measurement model in this study. However, maximum likelihood estimation is known to produce distorted results when the normality assumption is violated (Curran, West, & Finch, 1996). Multivariate normality was assessed using the Mardia measure of multivariate kurtosis (Mardia, 1970). The Mardia's coefficient for the data in this study was 31.55, which is lower than the value of 168 computed based on the formula *p* (*p* + 2) where *p* equals the number of observed variables in the model (Raykov & Marcoulides, 2008). On this basis, multivariate normality of the data in this study was assumed.

In the CFA, the scale of the latent factor was set by fixing the variance of the latent factor equal to one. The hypothesized and alternative models were nested so that the model fit could be compared between models using chi‐square difference tests. Moreover, as presented in Table 2, several indexes were used to determine the goodness of fit. The comparative fit index may range from 0 to 1, and values equal to or greater than 0.90 indicate a good fit to the data (Bentler & Bonnett, 1980; Kline, 1998). Similarly, scores 0.95 or above are desired with the Tucker–Lewis Index (close fit = 0.95–0.99, acceptable fit = 0.90–0.95, Bentler & Bonnett, 1980), the goodness of fit index, and the incremental fit index. Finally, a value of 0.08 or less for the root mean square error approximation reflects a model with an adequate fit to the data, whereas values greater than 0.10 strongly suggest that the model fit is unsatisfactory (Browne & Cudeck, 1989, 1992).

**Table 2:** Alternative models and significance test

Model	χ^2^	*df*	χ^2^/*df*	Δχ^2^	Δ*df*	GFI	CFI	IFI	RMSEA
1: One‐factor	109.17	71	1.54			0.93	0.95	0.95	0.05
2: Three‐factor	67.76	51	1.27	41.41	20	0.95	0.97	0.97	0.04

*Note*: Analysis is based on *N* = 336. Model 1 has one factor (Chikui). Model 2 has three factors (Chikui for conscience, Chikui for wealth, and Chikui for reputation). GFI, goodness of fit index; CFI, comparartive fit index; IFI, incremental fit index; RMSEA, root mean square error approximation.

The measures of fit for the different models are shown in Table 2. Model 2 (three‐factor) provided a better fit to the data than did Model 1 (one‐factor) (Δχ^2^ = 41.41; Δ*df* = 20, *p* < 0.01). The correlations among three factors were significant, ranging from 0.25 to 0.40 (*ps* < 0.01).

#### Reliability

Cronbach's alpha coefficients were calculated to estimate the reliability of the CLS. The internal consistencies were acceptable; Cronbach's alpha for the full scale was 0.79, and the alphas for the subscales ranged from 0.61 to 0.73.

After 1 month, we retested this scale with a sample of 92 undergraduates (58 women, 63.04%), and the retest coefficients of the three subscales were 0.83 (Chikui for conscience), 0.70 (Chikui for wealth), and 0.64 (Chikui for reputation). The retest coefficient of the total scale was 0.86. According to Hair, Babin, Money, and Samouel (2003), the reliability of the scale is acceptable.

#### Validity

##### Criterion‐related validity

Considering that the conceptual similarity between the present CLS and the “worth‐based choices” scale developed by Tang, Zhou, Zhao, and Li (2014) is, to our best knowledge, the closest, we decided to select the “worth‐based choices” scale as related criterion and to examine the criterion‐related validity of the CLS by assessing its relationship with the “worth‐based choices” scale.

Tang et al.'s (2014) scale is an 18‐item measure of “worth‐based choices.” There are four factors underlying these “worth‐based choices”: 惠 (favor), 善 (virtue), 义 (righteousness), and 法 (law). The following two sample items are from this scale:

1. If you have three apples, which option would you prefer? (A: eat all three apples by yourself, B: share two apples with a colleague or classmate)
2. Suppose there are two internships for you to choose from: one is a small private enterprise with a monthly salary of 3000 yuan; the other is a large foreign‐funded enterprise with a monthly salary of 2000 yuan. Which option would you prefer? (A: private enterprise, B: foreign‐funded enterprise)

It is apparent that the items in Tang et al.'s (2014) scale are a choice between a pair of offered options, while the item in the CLS is a single option to accept or not. In addition, the items in the CLS are borrowed from anecdotes of real‐world celebrities who are successful later, whereas items in Tang et al. (2014) are not.

The correlations between Tang et al.'s scale (2014), the CLS, and their subscales are presented in Table 3. Overall, we found a moderate correlation between Tang et al.'s scale (2014) and the CLS and their subscales. However, the law subscale of Tang et al.'s scale (2014) showed no significant correlation with the CLS and its subscales. The Chikui for the wealth subscale of the CLS did not correlate with the virtue and the righteousness subscales of Tang et al.'s scale (2014).

**Table 3:** Correlations between the Chikui Likelihood Scale, Tang et al.'s (2014) scale and their subscales (*N* = 200)

		Tang et al.'s (2014) scale				
		Total scores	Favor (惠)	Virtue (善)	Righteousness (义)	Law (法)
Chikui Likelihood Scales	Total scores	0.44***	0.36***	0.24***	0.26***	0.08
	Chikui for conscience	0.43***	0.25***	0.25***	0.29***	0.08
	Chikui for wealth	0.23**	0.30***	−0.01	0.12	0.06
	Chikui for reputation	0.22***	0.22***	0.28***	0.13*	0.03

* *p* < 0.05.

** *p* < 0.01.

*** *p <* 0.001.

##### Empirical and incremental validity

Our hypothesis regarding the empirical validity was that the “Chikui” likelihood scores would be greater for those who chose “Chikui” than those who did not. Considering that volunteers sacrifice their time and energy for the benefit of their communities, Tang et al. (2014) selected three types of volunteers (teaching in a remote area, serving at summer universiade, and donating blood without compensation) as people who chose “Chikui.” Their results revealed that volunteers' scores (*M*_teaching in a remote area_ = 78.03, *M*_serving at summer universiade_ = 74.68, *M*_donating blood without compensation_ = 70.89) of “worth‐based choices” were significantly higher than those of non‐volunteers (*M*_not teaching in a remote area_ = 71.58, *M*_not serving at summer universiade_ = 72.99, *M*_not donating blood without compensation_ = 67.96) (*ps* < 0.05). In the present study, we followed this logic and selected volunteers as people who chose “Chikui” to examine the empirical validity of the CLS. Moreover, considering that the correlations between Tang et al.'s scale (2014) and the CLS are moderate and that the “worth‐based choices” scale may serve as the most meaningful competitor for the CLS, we examined whether it is possible for the CLS to predict the outcome of volunteers after controlling for the scores of the “worth‐based choices” scale.

The result of the *t*‐test showed that volunteers had higher CLS scores (*M*_volunteers_ = 718.27) than non‐volunteers (*M*_non‐volunteers_ = 670.70) (*t*(198) = 2.21, *p* < 0.05). This result provided evidence in support of our hypothesis regarding the empirical validity. A binomial logistic regression analysis was then conducted with volunteers as the dependent variable, scores of the CLS as the independent variable, and participants' age, gender, and the scores of the worth‐based choice scale as the controlling variables. After controlling the variables mentioned earlier, CLS scores were significant incremental predictors of volunteers (Exp(β) = 1.002, Wald statistic = 4.236, *p <* 0.05) (Table 4). These results indicated that the CLS possessed good incremental validity when predicting volunteers.

**Table 4:** Results of binomial logistic regression analysis predicting volunteers (*N* = 200)

Model	Predictor variables	Volunteers	
		Wald	Exp(β)
Block 1	Age	2.651	0.710
	Gender	0.344	0.826
	Worth‐based choice scale	0.757	1.016
Block 2	CLS	4.236*	1.002

*Note:* Wald, wald statistic with a chi‐square distribution and one degree of freedom; Exp(β), exponent of the estimated coefficient; 95% CI = 95th percentile for the exponent of the estimated coefficient; CLS, Chikui Likelihood Scale.

* *p <* 0.05.

** *p <* 0.01.

Part 1 of this study represents an initial attempt to develop a CLS. Our findings concerning the test–retest reliability, criterion‐related validity, as well as empirical and incremental validity showed that the CLS is adequately valid and reliable for assessing people's tendency to choose “Chikui.”

### Part 2

The results of Part 1 provided initial reliability and validity data and support for the “Chikui” likelihood scale. Part 2 was then designed to provide further evidence regarding convergent, discriminant, criterion‐related, and incremental validity by employing a different sample.

Sacrificing money, time, or other valuable resources to help someone else or the community could be considered an important validity criterion of “Chikui” behavior. Nevertheless, these kinds of behaviors that look like “Chikui” behavior have been studied extensively and accounted for by certain personality traits (e.g., neuroticism or conscientiousness), sociopolitical attitudes, altruism, delayed gratification, and so forth. If the CLS measures meaningful constructs, then it should demonstrate convergent, discriminant, and incremental validity by a predictable pattern of relationships with other relevant variables within the “nomological network” (Cronbach & Meehl, 1955). Our hypotheses about the relationship between the CLS and relevant variables were as follows:

##### Convergent validity

We assumed that CLS scores would moderately correlate with the following variables: altruism (dictator game, DG), certain personality traits (e.g., neuroticism and conscientiousness), grit, delayed gratification, and social and political attitudes.

##### Discriminant validity

We expected that CLS scores would not correlate with participants' gender or academic performance.

##### Criterion‐related validity

To assess the relationship between the CLS and decision behaviors (rather than existing scales), we measured the behavioral decision outcomes to further examine the criterion‐related validity of the CLS. We expected that CLS scores would correlate with participants' choice behaviors in the ultimatum game (UG), DG, and intertemporal choice.

##### Incremental validity

It was important to provide evidence that the CLS displayed incremental validity over an established instrument that might explain “Chikui” behavior. That is, the CLS should capture unique variance in “Chikui” behavior that was not accounted for by relevant instruments when predicting real “Chikui” behavior. We expected that CLS scores would be a significant incremental predictor of volunteers and participants' willingness to have more than one child, after controlling the relevant variables within the “nomological network.”

### Method

#### Participants

Here, 296 undergraduate students (148 women, 148 men) were recruited from Shandong Normal University. The mean age of the participants was 19.41 years (*SD* = 0.75). Participants completed all measures individually and received a gift valued at ¥15 for their participation.

#### Materials and procedure

Participants completed the neuroticism and conscientiousness subscales (containing 17 items) drawn from the Chinese version of the Big Five Inventory‐44 (John, Donahue, & Kentle, 1991; John, Naumann, & Soto, 2008). Altruism was assessed with the DG, and the threshold for unfairness was measured by the UG (Forsythe, Horowitz, Savin, & Sefton, 1994; Thielmann & Hilbig, 2017). The mean UG scores were calculated using willingness circled on a 6‐point scale, and dictator allocations of 100 yuan made by participants in DG were coded. Grit was assessed with the Chinese version of the Grit Scale, containing 12 items rated on a 5‐point scale from 1 (not at all like me) to 5 (very much like me) (Duckworth, Peterson, Matthews, & Kelly, 2007). Delayed gratification was assessed with the Chinese version of the Academic Delay of Gratification Scale (ADOGS) containing 10 items rated on a 4‐point scale (Bembenutty & Karabenick, 1998). The Chinese version of the Social Dominance Orientation Scale (SDO) measured social and political attitudes using a 14‐item scale (Pratto, Sidanius, Stallworth, & Malle, 1994). Mean scores were calculated from responses to a 7‐point Likert scale. In addition, fixed‐sequence choice titration was measured, and the degree of discounting was calculated by application of the hyperbolic equation (Hardisty & Weber, 2009; Mazur, 1987). As in Part 1 of this study, the CLS was measured, and participants then answered questions about whether they were volunteers and their willingness to have more than one child. Participants also reported their gender, age, and academic performance (e.g., GPA) in the latest tests.

### Results and discussion

#### Convergent validity

The correlations of the CLS and other scales we measured are reported in Table 5. The CLS and its three subscales showed moderate positive correlations with the Grit Scale and conscientiousness on the BFI, with the correlation coefficients ranging from 0.12 to 0.21 (*p*s < 0.05). This suggested that participants who scored higher on the CLS and its subscales were more perseverant and conscientious. Furthermore, the CLS scale and its subscales were moderately negatively correlated with the SDO, with the correlation coefficients ranging from −0.19 to −0.26 (*ps* < 0.05). The “Chikui for reputation” subscale of the CLS showed negative correlations with the ADOGS. This indicated that participants scoring higher on the CLS were not likely to be discriminatory, and those who scored higher on the “Chikui for reputation” subscale tended not to delay gratification. However, CLS scores were not correlated with allocating more money to the recipient in the DG. This suggested that the CLS and altruism may be two distinct constructs.

**Table 5:** Correlations between the CLS and measures of related constructs (*N* = 297)

Measure (*N*)	*M*/*SD*	Alpha	CLS	Chikui for conscience	Chikui for wealth	Chikui for reputation
Neuroticism on the BFI	26.20/2.84	0.81	0.02	0.04	0.03	−0.03
Conscientiousness on the BFI	31.67/2.95	0.79	0.15*	0.18**	0.12*	0.01
Grit Scale	37.68/5.95	0.75	0.19**	0.21**	0.07	0.13*
ADOGS	28.50/3.89	0.66	0.04	0.09	0.06	−0.13*
SDO	44.83/11.48	0.86	−0.21***	−0.26***	0.03	−0.19***
Altruism (DG)	48.76/9.50		0.07	0.08	0.04	0.02
Gender^a^	0.49/0.50		0.01	0.07	−0.10	0.03
Academic performance (*n* = 142)			0.13	0.00	0.21	0.04

*Note:* DG, dictator game, BFI, Big Five Inventory, ADOGS, Academic Delay of Gratification Scale; SDO, Social Dominance Orientation; CLS, Chikui Likelihood Scale.

a 1, male; 0, female.

* *p <* 0.05.

** *p <* 0.01.

*** *p <* 0.001.

#### Discriminant validity

Table 5 shows the correlations between the “Chikui” likelihood scale, gender, and academic performance. In support of the CLS's discriminant validity, neither the CLS nor its subscales were correlated with participants' gender or academic performance.

#### Criterion‐related validity

In Part 1 of the present study, the criterion‐related validity was examined by analyzing the relationship between the CLS and the “worth‐based choices” scale developed by Tang et al. (2014). In Part 2, we sought to examine the criterion‐related validity of the CLS by assessing its relationship with participants' choice behavior in the UG, DG, and intertemporal choice rather than existing scales (Table 6). We found that the CLS correlated significantly with willingness to accept unfair allocations in the UG (*r* = 0.20, *p* < 0.01). The result suggested that individuals who were more willing to choose “Chikui” (suffering a loss) would be more likely to accept an unfair offer, which supported the criterion‐related validity of the CLS. However, the CLS was not correlated with allocating more money to the recipient in the DG (*r* = 0.07, *p =* 0.21). This finding, together with the correlations of CLS's subscale and the DG reported in Table 6, suggested that what is measured by allocating more money in the DG is decision makers' altruism and that the threshold of unfairness measured by willingness to accept unfair allocations in the UG was more related to “Chikui” than to altruism. Furthermore, CLS scores were not correlated with the degree of discounting in intertemporal choice. This may be because, in order to reach a decision, decision makers must only assess the outcomes of a pair of offered intertemporal options (“small but sooner” and “large but later”) on the fixed dimensions, while they have to generate a new dimension and assign a delayed subjective value (utility) to the single option of “Chikui.”

**Table 6:** Correlations of the CLS with UG, DG, and intertemporal choice

Measures	CLS	UG	DG	Intertemporal choice
CLS	1			
UG	0.20**	1		
DG	0.07	−0.00	1	
Intertemporal choice	0.04	0.08	0.03	1

*Note*: CLS, Chikui Likelihood Scale; DG, dictator game; UG, ultimatum game.

* *p* < 0.05.

** *p* < 0.01.

#### Empirical and incremental validity

Considering that volunteers who sacrificed time and energy to help others were taken as indicators of Chikui behavior both in Tang et al. (2014) and Part 1 of our study, we attempted to utilize it as an indicator to assess the empirical validity of the CLS in Part 2 Part 2 (which used a different sample). Moreover, considering that raising a child can cost parents a great deal of money, time, and energy and can be exhilarating and exhausting, participants who were willing to have more than one child were chosen to serve as another indicator of the empirical validity. To support the unique empirical validity of the CLS construct, some covariates that are of relevance to the “Chikui” construct, such as altruism and conscientiousness, were included to account for volunteers and participants who were willing to have more than one child.

The analysis of empirical validity in Part 1 of this study was repeated using another sample. The inclusion of this analysis also served to address the increased awareness of the need to replicate novel and important results (Baker, 2016). The results showed that volunteers had significantly higher CLS scores than non‐volunteers, which replicated the finding of Part 1 of this study (Table 7). These results provide repeated evidence in support of the empirical validity of the CLS.

**Table 7:** Comparing means of groups on the CLS

Group	*N*	*M*	*SD*	*t*	*p*
Volunteers	185	735.83	145.02	2.53	0.012
Non‐volunteers	109	688.76	167.78		
Participants who were willing to have one child	152	689.36	150.67	−3.25	0.001
Participants who were willing to have more than one child	143	747.34	155.65		

In addition, the results of the added indicator (participants who were willing to have more than one child) showed that participants who were willing to have more than one child had significantly higher CLS scores than participants who were only willing to have one child (Table 7). These results are additional evidence that the CLS exhibits good empirical validity.

A binomial logistic regression analysis was then conducted with volunteers and participants who were willing to have more than one child as the dependent variable, the score of CLS as the independent variable, and participants' age, gender, and conscientiousness score on the BFI, neuroticism score on the BFI, Grit Scale score, ADOGS score, SDO score, altruism DG, UG, and intertemporal choice as controlling variables (Table 8). After controlling the variables mentioned earlier, CLS scores were a significant incremental predictor of volunteers (Exp(β) = 1.002, Wald statistic = 4.163, *p <* 0.05) and participants who were willing to have more than one child (Exp(β) = 1.002, Wald statistic = 5.685, *p <* 0.05). The results indicated that the CLS possessed good incremental validity when predicting volunteers and participants who were willing to have more than one child.

**Table 8:** Results of binomial logistic regression analysis predicting volunteers and participants who were willing to have more than one child

Model	Predictor variables	Volunteers		Participants who were willing to have more than one child	
		Wald	Exp(β)	Wald	Exp(β)
Block 1	Age	5.471*	0.645	0.514	0.879
	Gender	2.120	1.555	3.500	1.728
	Conscientiousness on the BFI	0.051	1.012	6.736**	1.140
	Neuroticism on the BFI	1.281	1.058	0.000	1.000
	Grit Scale	0.144	1.009	0.212	1.011
	ADOGS	1.485	1.048	0.230	1.018
	SDO	0.051	1.003	0.172	1.005
	Altruism (DG)	2.528	1.025	0.079	1.004
	Ultimatum game	0.000	0.999	1.078	1.118
	Intertemporal choice	0.038	0.937	0.524	0.791
Block 2	CLS	4.163*	1.002	5.685*	1.002

*Note:* Wald, wald statistic with a chi‐square distribution and one degree of freedom; Exp(β), exponent of the estimated coefficient; 95% CI = 95th percentile for the exponent of the estimated coefficient. DG, dictator game, BFI, Big Five Inventory, ADOGS, Academic Delay of Gratification Scale; SDO, Social Dominance Orientation; CLS, Chikui Likelihood Scale.

* *p <* 0.05,

** *p <* 0.01.

The “Chikui” construct looked likely to be related to altruism, certain personality traits, and social values. To examine whether developing a new scale to explain decision‐making phenomena was meaningful, we assessed the validity of the CLS. The result indicated that the CLS accounted for “Chikui” phenomena above and beyond existing relevant constructs and measures, which meant that it provided an incremental contribution.

In all, the results we obtained suggested that the CLS was internally consistent and distinct from existing relevant measures.

## Study 2: The Current Relationship between “Chikui” Likelihood and Material or Mental Benefit

Study 2 was performed to determine whether there was a linear correlation between “Chikui” likelihood and real benefit. In our preliminary investigation, it was found that when asked to indicate whether they agreed that “suffering a loss is good fortune” on a 6‐point scale (1 = fully disagree; 6 = fully agree), the young Chinese surveyed tended to agree with the dogma, although members of older generations (supermarket customers, *N* = 88, 49 women, age over 40 years) agreed more than the young generation (undergraduate students, *N* = 92, 62 women, age range: 18–23 years; *M*_supermarket customers_ = 4.76, *M*_undergraduate students_ = 4.14, *t* = 4.1, *p* < 0.001). It was reasoned that the hidden goal of choosing Chikui (suffer a loss) in the present was to receive more material or mental benefit years later in an unpredictable life situation. Given that obtaining more material benefit requires some time to accumulate, someone who chooses Chikui at a younger age is more likely to gain more in his/her older years. Thus, we hypothesized that age would moderate the association between Chikui likelihood and real benefit. To test the moderation hypothesis, we set up two moderated multiple regression models, each consisting of one independent variable (Chikui), one moderator variable (age), and one outcome variable (material benefit or mental benefit).

The independent variable (Chikui likelihood) was measured by the CLS. The material benefit variable was measured using the Socioeconomic Index (SEI; Blau & Duncan, 1967; Duncan, 1984; Hauser & Warren, 1997), which is a widely used indicator of occupational ranking and based on education and income data from the 1950 census (Stevens & Featherman, 1981). The other variable, mental benefit, was measured by subjective well‐being (SWB; Andrews & Withey, 1976; Lu, 1995), which refers to how people evaluate their lives and includes variables such as life satisfaction (Zheng, Sang, & Lin, 2004).

### Method

#### Participants and procedure

The participants were 559 (363 women, 64.9%) adults who came from 20 Chinese provinces and were engaged in over 50 different occupations. The average age of the participants was 32.1 years (*SD* = 9.59, range = 20 to 74). Paper‐and‐pencil questionnaires were completed by 367 (65.65%) participants in the waiting lounge of the Ji'nan West Railway Station, and the other 192 (34.35%) participants completed an e‐questionnaire distributed through email. Each participant completed the scales independently and was given a small gift for their participation.

#### Measures

##### Chikui likelihood

We utilized the CLS, which we developed in Study 1, to assess participants' Chikui likelihood. Cronbach's alpha was 0.75.

##### Socioeconomic Index

It has been argued that the SEI for Western countries is not applicable for China (Li & Song, 1998; Xu, 2000); therefore, we used a revised Chinese SEI proposed by Li (2005), which has been shown to be efficient in China (*R* ^2^ = 0.81) to compute participants' SEI. The revised Chinese SEI formula is as follows:

SEI = 11.808 + 3.349 × length of education + 0.573 × average monthly income (hundred yuan) + 16.075 × top‐level managers 1A manager is the person responsible for planning and directing the work of a group of individuals, monitoring their work, and taking corrective action when necessary. According to their different hierarchical levels in an organization, managers can be classified as top‐level managers, middle managers, and low‐level managers (Katz, 1974). + 11.262 × middle‐level managers + 3.738 × low‐level managers + 8.942 × party and government offices + 6.841 × public institutions − 5.694 × enterprises − 26.655 × discriminated occupation.

##### Subjective well‐being

SWB was assessed by the following question: “How dissatisfied or satisfied are you with your life overall?” Answers to this question ranged from 1 = “not satisfied at all” to 6 = “completely satisfied.” This question was borrowed and modified from the Life Satisfaction scale (Campbell, 1976), which is the most commonly used measure in the SWB literature (Dolan, Peasgood, & White, 2006; Marsh & Bertranou, 2012).

#### Control of common method biases

To control common method biases, in the questionnaire design, we used the Podasakoff, MacKenzie, Lee, and Podsakoff (2003) method as follows: (i) participants complete the questionnaire anonymously, and facilitators ensure the subjects know that the survey is only for group study, not for individual analysis; (ii) each part of the questionnaire measures different points and has different scoring rules; (iii) each part of the questionnaire has a different reaction statement. Some have a probability from 0 to 100, some are in agreement from 1 to 6, and some are fill‐in‐the‐blanks.

In addition, Harman's single‐factor test (Harman, 1976; Podasakoff et al., 2003) was used to check for the potential for common method bias. Harman's single factor test is a widely used technique to diagnose common method variance (e.g., Andersson & Bateman, 1997; Aulakh & Gencturk, 2000; Greene & Organ, 1973; Krishnaveni & Deepa, 2013; Schriesheim, Kinicki, & Schriesheim, 1979). Therefore, we entered all the variables in the study into exploratory factor analysis using unrotated principal component factor analysis and principal component analysis with varimax rotation to determine the number of factors that are necessary to account for the variance in the variables. The factor analysis revealed the presence of six distinct factors with eigenvalues greater than 1.0 rather than a single factor. The six factors accounted for 59.45% of the variance, and the largest factor was found to account for only 17.7% of the variance. Thus, no general factor was apparent. According to the two criteria, there was no common method bias problem in this study.

### Results and discussion

#### Preliminary analyses

The summary statistics (i.e., means, standard deviations, Cronbach's alphas, and intercorrelations) for the variables under study are shown in Table 9. All measures demonstrated an acceptable level of internal consistency (0.78 < Cronbach's alpha <0.90).

**Table 9:** Means, standard deviations, Cronbach's alphas, and correlations between variables

Variables	*N*	*M*	*SD*	1	2	3	4
1. CLS	559	744.92	185.87	(0.79)^a^			
2. SEI^b^	542	89.80	24.37	0.29***	—		
3. SWB^c^	546^b^	4.84	1.00	0.21***	0.08	(0.90)^a^	
4. Age	559	29.30	7.48	0.24***	0.42***	−0.05	—

*Note*: CLS, Chikui Likelihood Scale; SEI, socioeconomic index; SWB, subjective well‐being.

a Reliability estimates (Cronbach's alphas) are in parentheses.

b Data of the SEI have 17 missing values.

c Data of the SWB have 13 missing values.

* *p* < 0.05.

** *p* < 0.01.

*** *p* < 0.001.

#### Moderating effect of age

Following the standard procedures for estimating and probing interaction effects outlined by Aiken and West (1991), we conducted hierarchical regression analyses to test our hypotheses. We first centered all predictors to reduce potential multicollinearity and increase interpretability. Then, the variables were included in the regression equation through three steps using spss. In Step 1 of the equation, we entered control variables such as gender, education years, and working years. In Step 2, we entered the two centered predictor variables. In Step 3, we entered the interaction term of the centered predictors to test for the presence of an interaction between the predictors.

The results indicated that the CLS score was positively and significantly correlated with the SEI (β = 0.21, *p* < 0.001) and SWB (β = 0.25, *p* < 0.001; Table 10). The CLS score explained 7.9% of the SEI variance and 5.8% of the SWB variance. This result supported our hypothesis that there would be a linear correlation between CLS scores and real benefits.

**Table 10:** Moderating effect of age

Model	Predictor variables	SEI			SWB		
		Model 1	Model 2	Model 3	Model 1	Model 2	Model 3
Step 1	Control variable						
	Gender	−0.22***	−0.13***	−0.14***	0.07	0.08*	0.07
	Education years				0.19***	0.16***	0.16***
	Working years				−0.04	−0.11	−0.13
Step 2	Independent variable						
	Chikui^a^		0.21***	0.19***		0.25***	0.25***
	Age^a^		0.34*	0.33**		−0.01	−0.02
Step 3	Chikui × age^a^			0.12***			0.04
*R* ^2^		0.05	0.23	0.24	0.05	0.11	0.11
Δ*R* ^2^		0.05	0.18	0.01	0.05	0.06	0.00
*F*		27.55***	55.39***	44.79***	8.49***	11.82***	9.96***
Δ*F*		27.55***	66.04***	10.21***	8.49***	16.41***	0.70

*Note:* SEI, socioeconomic index; SWB, subjective well‐being.

a Standardized regression coefficient.

* *p* < 0.05.

** *p* < 0.01.

*** *p* < 0.001.

The results presented in Table 10 indicate that age significantly moderated the relationship between CLS score and SEI (β = 0.12, *p* < 0.001). These moderating effects explained 1.4% of the SEI variance. 2We acknowledge that the effect should be considered small. However, the results revealed that age did not moderate the relationship between CLS score and SWB (β = 0.04, *p* > 0.05).

**Figure 2:**
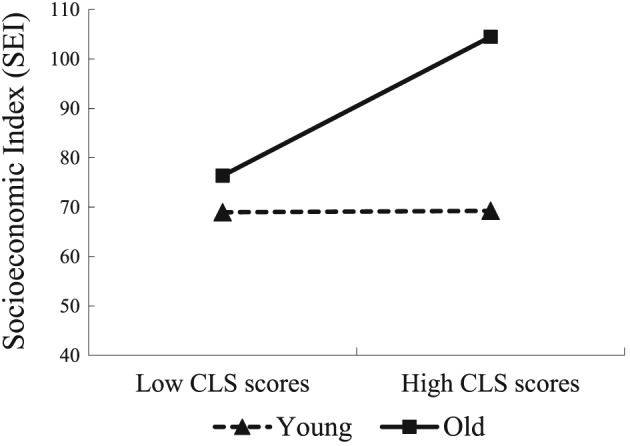
Relationship between CLS scores and SEI for young and old people. CLS, Chikui Likelihood Scale

To interpret the significant moderating effects, it was preferable to graphically represent them according to the procedure suggested by Preacher, Curran, and Bauer (2006). This procedure consists of calculating the regression equations involving the independent variables (CLS score) and the dependent variable (SEI) according to the low and high levels of the moderating variable (age), which correspond to one standard deviation below the average and one standard deviation above the average, respectively. Figure 2 demonstrates that the relationship between CLS score and SEI grew stronger as age increased.

In addition to plotting the moderating effects, we conducted simple slope analyses (Preacher et al., 2006). The simple slope of the regression of SEI onto CLS scores was significant when age was high (β = 0.07, *t*(540) = 9.35, *p* < 0.001) and nonsignificant when age was low (β = 0.01, *t*(540) = 1.60, *p* > 0.05). Therefore, the results of the simple slope analyses partly corroborated the hypothesis that age would moderate the association between Chikui likelihood and real benefits (Figure 2).

In this study, we utilized the CLS to determine whether there was a linear correlation between CLS scores and real benefits (either material or mental). The resulting findings revealed that CLS scores could positively predict SEI and SWB, suggesting that people who are more likely to choose Chikui are more likely to be satisfied with their life overall and to receive more material rewards. It is therefore relatively safe to say that “suffering a loss is good fortune” is not a myth but reality.

Table 10 shows the regression results. Most importantly, the predicted two‐way interaction was significant in the analysis of SEI. Simple slope analyses were conducted to illustrate the nature of the two‐way interaction (Aiken & West, 1991). More specifically, the CLS was more positively related to SEI as people aged. However, this effect was not observed between CLS scores and SWB. This result indicated that age moderated the relationship between Chikui likelihood and SEI but not the relationship between Chikui likelihood and SWB.

## Study 3: The Reverse‐predictive Effect of CLS Scores on Later Material or Mental Benefits

Study 2 showed that the current relationship between CLS scores and material benefits (but not mental benefits) is stronger for older people than for younger people. One possible explanation is that a person's material gains must be accumulated over time, whereas mental benefits may not. To test this possibility, inspired by the finding that preschool children's *delay of gratification* can predict long‐term coping and adjustment (Funder, Block, & Block, 1983; Mischel, Shoda, & Peake, 1988), we wanted to explore whether CLS scores could predict a person's long‐term material or mental benefit. We conjectured that the time interval would moderate the relationship between CLS scores and material benefits more heavily than it moderates the relationship between CLS scores and mental benefits.

The ideal way to do this is to conduct a longitudinal study. However, this method was not possible within the time constraints of the study. Therefore, we tried an alternative way to collect supporting evidence. Instead of measuring people's “Chikui” likelihood first and then their material or mental benefit years later, we measured people's present material or mental benefit and then asked them to assess their CLS score from years ago. If there was a correlation between people's present material or mental benefit and their retrospective CLS score, we termed it a reverse‐predictive effect of the CLS score on later material or mental benefit. Study 3 attempted to investigate this possibility. There was some empirical evidence demonstrating that the retrospective method we intended to use was effective (Bailey, Nothanagel, & Wolfe, 1995; Melamed, 1993; O'Gorman, 1982; Watson et al., 2013).

### Method

#### Participants

A total of three groups of college graduates were sampled. The first group comprised 1‐year alumni of Shandong Normal University; it included 142 alumni (99 women, 69.71%) aged 20 to 26 years (*M* = 22.89, *SD* = 1.23).

The second group comprised 10‐ to 13‐year alumni of Shandong Normal University; it included 171 alumni (112 women, 65.5%) aged 31 to 39 years (*M* = 34.01, *SD* = 5.61).

The third group consisted of 75 adults who graduated from university more than 20 years prior (30 women, 40%) and were aged 40 to 74 years (*M* = 49.56, *SD* = 8.46). The participants were adult passengers departing from Ji'nan West Railway Station, and the data collection took place in the waiting lounge of the railway station.

#### Measures

The measures employed in Study 2 were also used in this study. In addition to measuring each subject's current CLS score (i.e., the CLS score assessed for the present) as in Study 2, we measured their imagined past CLS score (i.e., the CLS score recalled from the past). The instruction for recalling the “CLS score for the past” was given as follows:

“For each scenario, please write 2 values: one that reflects the likelihood that you would do the same thing as the person described, and a second that reflects the likelihood that you personally would have done this when you were a college student.”

An example of the 24 scenarios measuring Chikui likelihood (Appendix S1) was as follows:

During an outbreak of SARS (severe acute respiratory syndrome), the food industry was severely affected. However, a restaurant owner did not lay off any employees and paid full salaries on time.

### Results and discussion

#### Preliminary analyses

**Figure 3:**
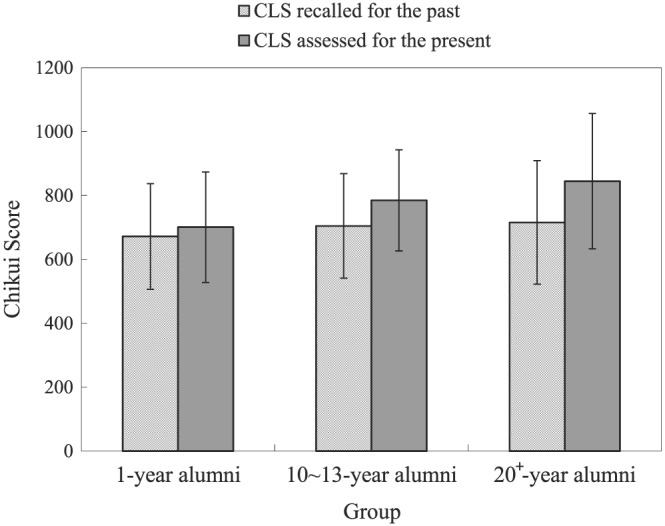
Comparison of the CLS assessed for the present with the CLS recalled for the past across three groups of college graduates (1‐year alumni, 10‐ to 13‐year alumni, 20+‐year alumni). CLS, Chikui Likelihood Scale

Table 11 shows the means, standard deviations, and intercorrelations among the variables used in Study 3. Figure 3 depicts the comparison of CLS scores assessed for the present and CLS scores recalled for the past. All variables were analyzed with two‐way ANOVAs with a repeated factor “CLS score” (present vs. past) and a between‐subject factor “group” (1‐year alumni, 10‐ to13‐year alumni, and 20+‐year alumni). The result revealed a main effect of CLS score (present vs. past), *F*(1, 385) = 199.69, *p* < 0.001. The CLS scores assessed for the present (764.29 ± 193.05) were higher than the CLS scores recalled for the past (683.64 ± 183.24). The main effect of group (1‐year alumni, 10‐ to 13‐year alumni, and 20+‐year alumni) was significant, *F*(2, 385) = 8.05, *p* < 0.01. Pairwise comparisons showed that both 10‐ to 13‐year alumni's CLS scores (present and past) and 20+‐year alumni's CLS scores (present and past) were significantly higher than 1‐year alumni's CLS scores (present and past). The results also showed a significant interaction effect between CLS score and group, *F*(2, 385) = 15.83, *p* < 0.001. A further simple effect analysis revealed that 1‐year alumni's CLS scores assessed for the present (705.80 ± 170.82) were lower than those of other groups (10‐ to 13‐year alumni (776.37 ± 159.51), 20+‐year alumni (848.84 ± 257.95)), *ps* < 0.001. However, CLS scores recalled for the past did not differ significantly among the three groups.

**Table 11:** Means, standard deviations, and intercorrelations among variables used in Study 3

Group	Variables	*N*	*M*	*SD*	1	2	3	4
I	1. SEI	142	83.48	5.98	—			
	2. SWB	142	5.05	0.77	−0.05	—		
	3. CLS assessed for the present	142	705.05	170.82	−0.09	0.22*	—	
	4. CLS recalled for the past	142	661.31	165.57	−0.06	0.15	0.70***	—
II	1. SEI	171	109.98	19.86	—			
	2. SWB	171	4.83	0.93	−0.02	—		
	3. CLS assessed for the present	171	776.37	159.51	0.23**	0.23**	—	
	4. CLS recalled for the past	171	692.82	165.41	0.10	0.19	0.75***	—
III	1. SEI	73	116.66	33.55	—			
	2. SWB	75	4.81	0.78	0.30**	—		
	3. CLS assessed for the present	75	848.84	257.95	0.68***	0.53***	—	
	4. CLS recalled for the past	75	708.81	242.40	0.65***	0.39**	0.79***	—

*Note:* Group I is 1‐year alumni. Group II is 10‐ to 13‐year alumni. Group III is 20+‐year alumni. SEI, socioeconomic index; SWB, subjective well‐being; CLS, Chikui Likelihood Scale.

* *p <* 0.05.

** *p <* 0.01.

*** *p <* 0.001.

Our preliminary investigation showed that the older generation agrees more with “suffering a loss is good fortune” than the younger generation, and Study 2 showed that individuals' CLS scores increased with age. Those findings were consistent with the present finding that the past CLS scores collected by the retrospective method were lower than the current CLS scores and that there was no significant difference in past CLS scores among the groups. This consistency to some extent implied that the retrospective method was reliable.

#### Reverse‐predictive effect of Chikui likelihood on SEI and SWB

**Figure 4:**
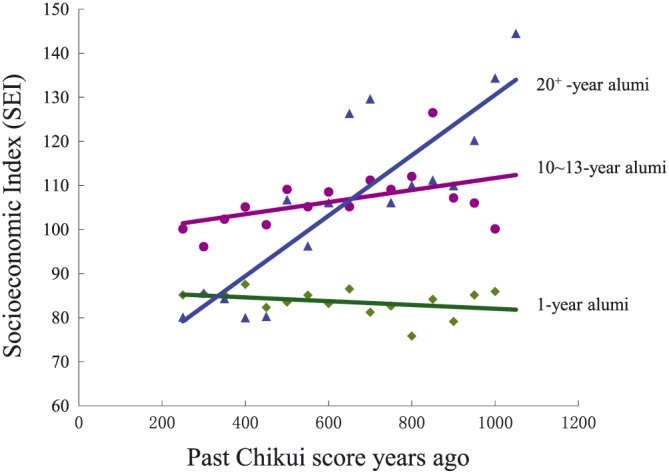
SEI (Social Economic Index) as a function of Chikui Likelihood Scale score recalled for the past. The lines correspond to the three groups of college graduates: 1‐year alumni (green), 10‐ to 13‐year alumni (purple), and 20+‐year alumni (blue) [Colour figure can be viewed at http://wileyonlinelibrary.com]

We used hierarchical regression to test the reverse‐predictive effect of CLS scores recalled for the past on SEI and SWB. The control variables were gender, age, education years, working years, and CLS scores assessed for the present. As shown in Table 12 and Figure 4, the CLS scores recalled for the past significantly positively predicted SEI (β = 0.46, *p* < 0.05; *ΔR* ^2^ = 0.03, *p* < 0.05) for 20+‐year alumni but did not predict current SWB for any of the three groups.

**Table 12:** Hierarchical regression analysis of CLS scores recalled for the past on SEI and SWB

Variable	SEI			SWB		
	1‐year alumni	10‐ to 13‐year alumni	20+‐year alumni	1‐year alumni	10‐ to 13‐year alumni	20+‐year alumni
Control variable						
Gender	−0.15*	−0.13*	−0.25**	−0.03	0.12	0.02
Age	−0.06	−0.02	−0.06	−0.02	0.12	−0.17
Education years				−0.03	0.06	−0.04
Working years					0.02	−0.06
Current CLS score	0.02	0.33**	0.29*	0.23*	0.19*	0.44***
Independent variable						
Past CLS score	−0.08	0.11	0.46*	−0.01	0.05	0.07
*R* ^2^	0.04	0.26	0.49	0.05	0.09	0.24
Δ*R* ^2^	0.01	0.01	0.03	0.00	0.00	0.00
*F*	1.26	4.55***	13.25***	1.41	2.63*	3.52**
Δ*F*	0.44	1.49	6.61*	0.01	0.23	0.06

*Note:* SEI, socioeconomic index; SWB, subjective well‐being; CLS, Chikui Likelihood Scale.

* *p* < 0.05.

** *p* < 0.01.

*** *p* < 0.001.

In short, utilizing a retrospective method to measure participants' CLS score from years past, we found that the current relationship between Chikui likelihood and material (but not mental) benefits could be extended when asking participants to recall and assess their retrospective CLS score from years past. That is, CLS scores recalled for the past were correlated with higher levels of SEI in the present. This effect was strengthened as the interval between the time point of the memories and the present increased. Apparently, the prediction of SEI from earlier past CLS scores presented a time lag and an accumulated effect. This finding is consistent with a remark by Zheng Banqiao: “Losers gain more gradually (亏者盈之渐).” However, this effect was not found in the relationship between Chikui likelihood and mental benefit.

In sum, Study 3 duplicated the result of Study 2. The current CLS score was positively related to SEI and SWB. Furthermore, Study 3 verified the conjecture derived from Study 2. That is, the positive effect of Chikui likelihood on SEI became more significant as people aged, but the effect of Chikui likelihood on SWB did not change with time. We found that CLS scores recalled for the distant past (over 20 years) could significantly positively predict SEI, but this was not the case for the near past (1‐year alumni and 10‐ to 13‐year alumni). This effect was not found in the relationship between Chikui likelihood and mental benefits, which suggests that mental reward is gained spontaneously.

## General discussion

To answer the question of whether the long‐lasting Chinese dogma “吃亏是福” (suffering a loss is good fortune) can provide a satisfactory account for taking a single option that will result in apparent loss in our modern society, we first developed an anecdote‐based scale to measure “Chikui” likelihood. The unique features of the scale are as follows: (i) each item that was supposed to measure “Chikui” was making a decision over whether to take a single option rather than choose from a pair of options, which was used in previous studies on suffering a loss (i.e., Tang et al., 2014); (ii) each “Chikui” item is a rephrased anecdote of celebrities before they were famous; and (iii) the “Chikui” likelihood was then measured by “walking in another person's shoes” (C. R. Rogers, 1902–1987), which means indicating the likelihood that a responder would act in the same way that was described.

Three factors (Chikui for conscience, Chikui for wealth, and Chikui for reputation) were extracted by an exploratory factor analysis, and the three‐factor construct was shown to be reliable and valid by the results of a CFA. Moreover, the convergent, discriminant, and incremental validity of the CLS were supported by analyzing the relationships between the CLS and other relevant variables within the “nomological network.” The result indicated that the developed CLS possessed incremental validity above and beyond existing relevant constructs and measures when accounting for “Chikui” phenomena.

We then found that CLS scores were higher for those who chose “Chikui” (e.g., volunteers or participants who were willing to have more than one child) than those who did not and that CLS scores could positively predict SEI and SWB. These results suggest that “Chikui” is somewhat like a competency that people need to be successful in their lives.

The good fortune (福) described in the dogma, in terms of material or mental benefits, was measured by SEI and SWB in the present study. As for mental benefits, our findings revealed a relationship between “Chikui” likelihood and mental benefit. That is, the more likely a person is to choose Chikui, the happier that person currently is. This is true regardless of age. This finding is consistent with what Zheng Banqiao said: “Miss a move, take a step back, for immediate peace of mind, not in the hope of later reward” 3放一着,退一步,当下安心,非图后来报也。The English version of this calligraphy is from Barme and Jaivin (1992, p. 439). (Barme & Jaivin, 1992).

Moreover, we found that Chikui likelihood was correlated not only with immediate peace of mind (mental benefit) but also with immediate or later reward (material benefit). Although Zheng Banqiao did not hope for a later reward to result from Chikui, we found a reverse‐predictive effect of “Chikui” likelihood on later material benefit only when the past CLS score was recalled from more than 20 years prior. The observation that the past CLS score that was recalled from more than 20 years prior was correlated with SEI is reasonable given that a person's material gains are accumulated over time. In short, our findings suggested that choosing Chikui can result in not only mental benefits but also material benefits, and not only immediate rewards but also later rewards if time permits. It can, therefore, be stated confidently that “suffering a loss is good fortune” is not a myth but a certain reality.

The revealed material or mental benefits are supportive to our conjecture that “there exists an extra dimension that is not offered but self‐generated dimension.” Otherwise, there will be no “gain” dimension in the space to represent the material or mental benefits we found and reported. The three factors (Chikui for conscience, Chikui for wealth, and Chikui for reputation) we generated by applying factor analysis are very likely to be a good candidate for any extra dimension (D_*j*_) (Figure 1b).

Using the option‐representing framework in Figures 1 as a basis, it would be interpretable and easy to make theoretical sense of the present findings. That is, *without* the extra dimension (D_*j*_) in mind, the decision maker had to assign a value (utility) to the option on the offered dimension(s) and obey the principle of value maximization (Figure 1a). Thus, when faced with a pair of choices, the decision maker's only choice is to behave according to the jungle law of “achieving rewards and avoiding punishments” or, when offered **a single option**, act as “When it was to their advantage, they made a forward move; when otherwise, they stopped still.” (*The Art of War* 4
*The Art of War* is a Chinese military treatise written by Sun Tzu during the 6th century bc.).

In contrast, *with* the extra dimension (D_*j*_) in mind, the decision maker will be able to assign a delayed subjective value to the option on the newly generated dimension. The material or mental benefits we found and reported are well above and beyond what is represented in a one‐dimensional or multidimensional space (Figure 1b). Once the value (utility) assigned to the option on the newly generated dimension (D_*j*_) is greater than that assigned to the option on the offered dimension (D_*i*_), to take an option that results in loss is no longer a puzzling “paradox” but a must for us to do in a very natural way.

We therefore reason that those who choose to “Chikui” (suffer a loss) are those who have assigned a greater delayed subjective value to the option on the newly generated dimension.

There were four potential limitations to our study. First, we only conducted a cross‐sectional study in Study 3, whereas it would be preferable to measure Chikui likelihood earlier and then track the material and mental benefit of our participants years later. In the absence of a longitudinal study, our findings are suggestive but do not prove causality, and the following two questions therefore remain unaddressed: (i) we are unable to confirm whether the correlation found in Study 2 means that it is the belief that boosts both financial and psychological well‐being, rather than the reverse (i.e., greater present material benefit may increase present “Chikui” likelihood). Only a longitudinal study can fully assess this relationship, and (ii) we cannot confirm why current Chikui likelihood was more predictive of SEI than recalled Chikui likelihood for the 10‐ to 13‐year alumni (the opposite of what we predicted). If a longitudinal SEI (rather than recalled Chikui likelihood) was measured a decade ago and the result was the opposite of what we predicted, the notion of “suffering a loss is good fortune” would be challenged and shown to be contradictory. However, the present data leave it an open question as to whether this is true or not.

Second, the measurement of mental benefit in our study was not a state‐of‐the‐art assessment. To shorten the length of our questionnaire, the Life Satisfaction Scale (Campbell, 1976), which is a concise measurement and the most commonly used measure in the SWB literature (Dolan et al., 2006; Marsh & Bertranou, 2012), was adopted in the present study. It would certainly be helpful if future studies were able to utilize a modern instrument such as the Gallup World Happiness Report to measure participants' mental benefits.

Third, considering the great length of time and number of measurements involved, not all subscales of the Big Five Inventory were measured to determine the convergent validity of the CLS. Thus, our conclusion regarding the correlation between the CLS and the Big Five Inventory may be inconclusive given that only the neuroticism and conscientiousness subscales of the Big Five Inventory, which we believe were relevant to the CLS, were assessed in the present study.

Last, although Studies 2 and 3 used Harman's single factor test to check for common method variance and the results suggested common method bias was unlikely to confound the results, it could be argued that this claim is likely to be incomplete because Harman's test is insensitive (Podasakoff et al., 2003). In future studies, using multiple sources for data collection would be helpful for minimizing this problem.

Taken together, our findings suggest that the traditional Chinese dogma “吃亏是福” (suffering a loss is good fortune) might play a “nudge” role in how objectives of a green economy and sustainable development can be achieved.

We are living in a rapidly changing environment (Wei, Tao, Liu, & Li, 2017). In the context of transition to a green economy and sustainable development, if our vision is strictly limited to the space where a single option that will result in apparent loss was represented by a fixed set of dimensions (e.g., any dimension that represents exploiting natural resource wealth), it is unlikely that the objectives of a green economy and sustainable development will be achieved.

In fact, at present, millions of Chinese are being faced with a single offered option that will result in apparent loss such as compress, suspend, or close a profitable family business or state‐owned enterprise. For instance, in Shandong Province, there are about six million livestock and poultry farms that would have been shut down or relocated if the livestock pollution requirements were not met before 2017 (Zhao, 2016). In addition, over one million fishermen (187,000 fishing boats) in China coped with the fishing moratorium from May 1 to September 1 in the East China Sea, the Yellow Sea, the Bohai Sea, and the South China Sea (Chu, 2017), and the steel industry in China will cut crude steel capacity by 100 to 150 million tons over the next 5 years (Lu, 2016).

To ensure success in the mission of shutting down the businesses that are still making money and that millions of people live on, the key issue is whether the decision maker and policy maker are aware of the extra (hidden green) dimension (D_*j*_), which represents the single option that will result in apparent loss. In this sense, efforts to enhance environmental protection consciousness (such as that proposed by the government or an NGO) do, in fact, emphasize that there is an invisible “green” dimension. The purpose of thinking green is to make it clear that choosing to “Chikui” (suffer a loss) on an offered dimension that represents exploiting natural resource wealth will gain something on an extra dimension that represents reducing environmental pollution and ecological impact. That an individual or organization, at present, should gain less, gain nothing, or even lose something (Chikui) in return for green and sustainable development in the future is exactly what the philosophy of “suffering a loss is good fortune” prescribes.

In sum, our findings offer insights into the relationship between short‐sighted self‐interest and long‐term perspectives in real‐world decision making. The decision‐making approach derived from “suffering a loss is good fortune” might enlighten policy makers and managers in state administration and promote green development and green lifestyles as the country seeks to balance economic growth and environmental protection. These findings also provide an empirical reference for young people who are entering society and must choose among survival rules for life.

## Supporting information

Appendix S1
