# Supplementary material for: Suffering a Loss Is Good Fortune: Myth or Reality?
Source: J Behav Decis Mak. 2017 Nov 29;31(3):324–40. doi: 10.1002/bdm.2056 (PMC6033005; doi:10.1002/bdm.2056)
Supplement: Supplementary file 1 — Appendix S1 [file BDM-31-324-s001.docx]

**Appendix**

**Please carefully read the following scenarios and, based on your personal experiences, enter values between 0% and 100% to indicate the likelihood that you would act in the same way that is described.**

**For each scenario, please write two values: one that reflects the likelihood that you would do the same thing as the person described and a second that reflects the likelihood that you would do the same thing if you were a college student.”** ^[[1]](#footnote-0)^*

1 Zhang went to a store to purchase some goods. After Zhang paid for the goods and went home, a representative from the store called and told Zhang that one of the 100-yuan bills was a counterfeit note, but the person was unsure whether the bill came from Zhang. Nevertheless, Zhang returned to the store and exchanged the money.

2 Li sold some carpet cleaning equipment to a hotel. After the equipment was delivered, the hotel asked to return the equipment, claiming that it was broken during delivery. Li sent someone to inspect the equipment and found that the damage was the result of improper assembly. Li believed that the company was not liable for the damage, but Li agreed to pay for the repair.

3 Wang is the owner of a small business and is in the process of discussing merchandise supply with a company in the Three Gorges region. If his business passed the inspection, the company agreed to allow Wang to submit a bid. The inspection would be conducted in 2 months by specialists from the company. Wang’s business did not have capital or a factory. In the expectation of a contract, he borrowed 3 million yuan and started building a factory.

4 A surgeon and his doctoral students, including Zhang, performed heart surgery on a patient. During the operation, the patient suddenly died on the operating table for unknown reasons. Some students were so frightened that they were weak at the knees. In contrast, Zhang calmly walked to the operating table and opened the heart to determine the cause of death.

5 A business executive was deciding on an important project that involved presenting to a government official in charge of a department. When the executive arrived in Beijing for the presentation, the official had gone to Shanghai; when the executive rushed to Shanghai, the official had gone to Shenzhen. The executive finally met with the government official after making 7 unsuccessful trips.

6 A host of a radio station was let go 18 times in her career; her hosting style was condemned as worthless. Nevertheless, she continued with persistence to look for her next hosting job.

7 An elderly couple arrived at a hotel on a stormy night and requested to stay because all the nearby hotels were fully occupied. “Our hotel is also full. But if you don’t mind, please take my bed!” said the clerk at the hotel. The next morning, when the couple tried to pay for the room, the clerk said, “My own bed is not for profit, so I will not take your money!”

8 When an elderly woman walked into a department store on a rainy day, most of the sales clerks ignored her. One young man asked her if she needed anything, and the woman responded by saying that she was only there to get out of the rain. The young man did not try to sell her anything and instead offered her a chair.

9 A newly established construction company finally acquired a project. Because many parts of the project were subcontracted, the net loss after the project was completed was 50,000 yuan. Nevertheless, the company owner said, “There’s nothing more important than ensuring the schedule and the quality of this project, even if we lose 80,000 yuan instead of 50,000 yuan”.

10 The leading product of a company can sell for 5,000 yuan per item, but the business owner sets the price at 3,000 yuan.

11 Li is a horticulturist who earns a monthly salary of 3,000 yuan. Someone is willing to invest 1 million yuan to start a business with him, putting him in charge of seedling management and selling the trees after 3 years, sharing profits and losses. Although Li’s family strongly protested the idea, Li resigned from his job and started the business.

12 To promote a desktop publishing system, with only 4,000 yuan left in his pocket, Sun booked an advertising space in *Computer World* for 8,400 yuan. He asked to publish the advertisement before making payment.

13 When a young lawyer first stepped into the field of law, his career was almost a complete failure. Regardless of his obstacles, he said, “I will strive for success 999 times, and if each result is a failure, I shall try for the thousandth time”.

14 Sun owns a secret recipe for a popular fried chicken dish. He has attempted to sell his secret recipe to many restaurants, hoping to strike a business opportunity to collaborate with a restaurant. Even after 1,000 rejections, he continues to search for the next restaurant.

15 After achieving success, Zheng is grateful not only to his supporters but also to his enemies.

16 A mother-in-law made life extremely difficult for her daughter-in-law. When the mother-in-law was diagnosed with the plague, everyone except for the daughter-in-law avoided her; the daughter-in-law voluntarily stayed and took care of her mother-in-law.

17 Shortly after the Second World War, when the economy was in a downturn, a family-controlled conglomerate in the U.S. donated land valued at 8.7 million dollars to the United Nations. The conglomerate also purchased the large area of land adjacent to the donated land.

18 As a result of a lack of understanding of various types of products, Zhang often stocked counterfeit products during the initial stage of running a computer business. He purchased products from other places to compensate his customers, and he hired technicians to offer after-sales maintenance, which he paid for out-of-pocket.

19 In the mid-1960s, the social condition in mainland China caused a widespread panic in Hong Kong, and many people tried to sell their houses at low prices. As a real estate developer, Li invested all of his funds in real estate acquisition.

20 Qian is a high school student who built a website when the internet first became popular. Qian earned 100,000 yuan from the website. A short time later, the internet bubble burst. Nevertheless, Qian decided not to take the college entrance examination and chose to start a business.

21 To realize his dream of building the “happiest place on earth”, Dai began to raise funds. However, every bank he visited refused to provide funds because they thought his idea was bizarre. After 300 rejections, Dai continued to search for the next bank.

22 Wang was optimistic about a brand-name underwear. However, after 2 years of promotion, even as the promotion fee decreased from 5 million to 100,000 yuan, none of the factories was willing to invest in and manufacture the underwear. The promotion team members left one by one, but Wang believed that demand was always there for good products; therefore, Wang insisted on continuing to promote the brand.

23 When a mineral water salesman was delivering water, he realized that the customer was an elderly woman in a wheelchair. The woman happened to have an out-of-town guest arriving that day and needed to pick up the guest. The salesman volunteered to help the woman pick up her guest.

24 During an outbreak of SARS (severe acute respiratory syndrome), the food industry was severely affected. However, a restaurant owner did not lay off any employees and paid full salaries on time.

1. * The respondents to respond to more than one option for each question, i.e., for Question 1:

   **Imagine that you were Zhang in this situation.** What is the likelihood that you would do the same thing? （ ）0%————————100%

   **Imagine that you were in college and facing this situation**. What is the likelihood that you would do the same thing? （ ）0%————————100% [↑](#footnote-ref-0)
